# Garlic Ecotypes Utilise Different Morphological, Physiological and Biochemical Mechanisms to Cope with Drought Stress

**DOI:** 10.3390/plants12091824

**Published:** 2023-04-28

**Authors:** Ivanka Habuš Jerčić, Anita Bošnjak Mihovilović, Ana Matković Stanković, Boris Lazarević, Smiljana Goreta Ban, Dean Ban, Nikola Major, Ivana Tomaz, Zrinka Banjavčić, Snježana Kereša

**Affiliations:** 1 Department of Plant Breeding, Genetics and Biometrics, Faculty of Agriculture, University of Zagreb, Svetošimunska cesta 25, 10000 Zagreb, Croatia; 2 Department of Plant Nutrition, Faculty of Agriculture, University of Zagreb, Svetošimunska cesta 25, 10000 Zagreb, Croatia; 3 Centre of Excellence for Biodiversity and Molecular Plant Breeding, Faculty of Agriculture, University of Zagreb, Svetošimunska cesta 25, 10000 Zagreb, Croatia; 4 Institute of Agriculture and Tourism, Karla Huguesa 8, 52440 Poreč, Croatia; 5 Department of Viticulture and Enology, Faculty of Agriculture, University of Zagreb, Svetošimunska cesta 25, 10000 Zagreb, Croatia

**Keywords:** *Allium sativum* L., drought, spectral characteristic, total phenolic content, soluble sugars, inulin, free amino acids, antioxidant activity

## Abstract

Drought negatively affects plants by altering morphological, physiological and metabolic processes and ultimately reducing yields. Garlic (*Allium sativum* L.), an important member of the Alliaceae family, is also sensitive to drought and maximizing the yield of garlic bulbs is largely dependent on water availability. The objective of this study was to determine the effects of drought stress on morphological and physiological characteristics, as well as on phenolic, sugar, inulin and free amino acid content and antioxidant activity in two Croatian garlic ecotypes, ‘Istarski crveni’ (IC) and Istarski bijeli (IB). Drought was induced by using polyethylene glycol 8000 (PEG) solution (−0.6 MPa) starting 21 days after clove planting and lasted for 20 days. Drought reduced plant height, number of leaves and plant weight, but increased root length in both ecotypes compared to the control treatment. Among the physiological parameters, significant differences were observed between the two ecotypes studied in the spectral characteristics of the leaves, namely reflection in red, green and blue, VAL, values of the vegetation indices related to the chlorophyll content (CHI, GI), and the anthocyanin content (ARI). Ecotype IC showed higher antioxidant activity in the control treatment due to higher total phenolic content (TPC), but under drought conditions higher DPPH radical scavenging activity was determined in ecotype IB and higher values of FRAP in IC. Sucrose and glucose generally decreased under drought, while inulin increased in IB but decreased in IC. Total free amino acid content increased under drought in both ecotypes. In conclusion, drought tolerance of IB might be associated with increased accumulation of inulin and higher levels of amino acids, especially those shown to contribute to drought resistance. In IC, drought tolerance is associated with an increase in some amino acid compounds and better root growth in depth, probably due to a more efficient translocation of sucrose to the underground part of the plant.

## 1. Introduction

With the advent of global climate change, plants are increasingly exposed to various abiotic stresses, including extreme temperatures and prolonged drought [1]. Drought is one of the most important limiting factors for agricultural production, leading to a significant reduction in crop yields worldwide [2,3]. Drought affects morphological, physiological, biochemical and molecular characteristics of plants [4,5]. Therefore, plants have evolved different adaptive mechanisms and strategies to increase tolerance to water shortage [4,6].

Drought can cause damage to photosynthetic organs, changes in cell function and structure, impaired metabolic function, reduced rate of absorption and transport of nutrients, inhibited plant growth, leaf chlorosis and wilting, disruption of phytohormone balance, increased plant energy consumption, reduced plant quality, shortened life span and even plant death [5,7,8,9,10].

One of the inevitable consequences of drought (and other stresses) is the increase in reactive oxygen species (ROS) levels [11]. ROS fulfil the role of signalling molecules to initiate the synthesis of enzymes and non-enzymatic molecules involved in antioxidant reactions [12], but excessive accumulation of ROS leads to lipid peroxidation, DNA damage, changes in protein structure and function, and eventually programmed cell death. However, these processes are mitigated in cells by various ROS detoxifying proteins [13] and the biosynthesis of secondary metabolites including polyphenols [14]. Phenolic compounds are an effective non-enzymatic protective agent against ROS species [15,16,17]. These compounds can serve as direct antioxidants by interacting with ROS species or as electron donors to enzymatic antioxidant systems such as peroxidases that effectively neutralize ROS species [17,18,19]. In plants, phenolic accumulation of phenolics is usually a consistent feature of plants under stress, representing a defence mechanism to cope with multiple abiotic stresses [19].

Under osmotic stress, the cell is trying to combat dehydration by biosynthesizing osmoprotectants, mainly soluble sugars, ammonium compounds and amino acids [20]. Drought not only inevitably triggers the accumulation of sugars in plants [10,21], but also promotes the breakdown of storage sugars (such as starch) into soluble sugars (such as sucrose, glucose, fructose, etc.), which consequently reduces the water potential of the cell [10,22]. The accumulation of amino acids generally increases in response to various abiotic stresses. Among drought-induced amino acids in plants, proline (Pro) has been studied most extensively [23]. Pro synthesis is strongly induced under osmotic stress; thus, increased Pro concentration can be used as a metabolic stress indicator [24]. While Pro is accumulated during osmotic stress through *de novo* biosynthesis, recent studies suggest that autophagy and abscisic acid-induced protein turnover contribute to the increase in other free amino acids [24]. This especially applies to branched-chain amino acids (BCAAs; leucine, isoleucine and valine), whose concentration in plants under drought is often higher than that of proline [23].

As a shallow-rooted plant, garlic (*Allium sativum* L.) is sensitive to water stress [25,26,27]. The effects of water deficit on garlic depend on its intensity and the growth phase in which it occurs [28]. Water deficit leads to reduced plant development, cell division and photosynthesis. Reduced photosynthesis leads to reduced leaf area index, resulting in reduced light absorption, photosynthetic area, dry matter and plant growth [26]. The bolting/bulbing stage is the most critical period when adequate water supply is very important [26]. In Croatia, mainly old varieties and ecotypes of garlic are cultivated. Since the region of Istria suffers from a lack of rainfall throughout the year, the ecotypes grown there have adapted to dry conditions. To determine the mechanisms of their drought tolerance, two ecotypes, ‘Istarski crveni’ and Istarski bijeli, were studied. ‘Istarski crveni’ is listed as a conservation variety on the List of Varieties of the Republic of Croatia, while Istarski bijeli is a local landrace and both are grown by farmers in Istria.

The aim of this study was to determine the effects of drought on morphological and physiological characteristics as well as on phenolic, sugar, inulin and free amino acid and antioxidant activity in two Croatian garlic ecotypes, ‘Istarski crveni’ (IC) and Istarski bijeli (IB).

## 2. Results and Discussion

### 2.1. Influence of Drought on Morphological Characteristics of Garlic

**Figure 1:**
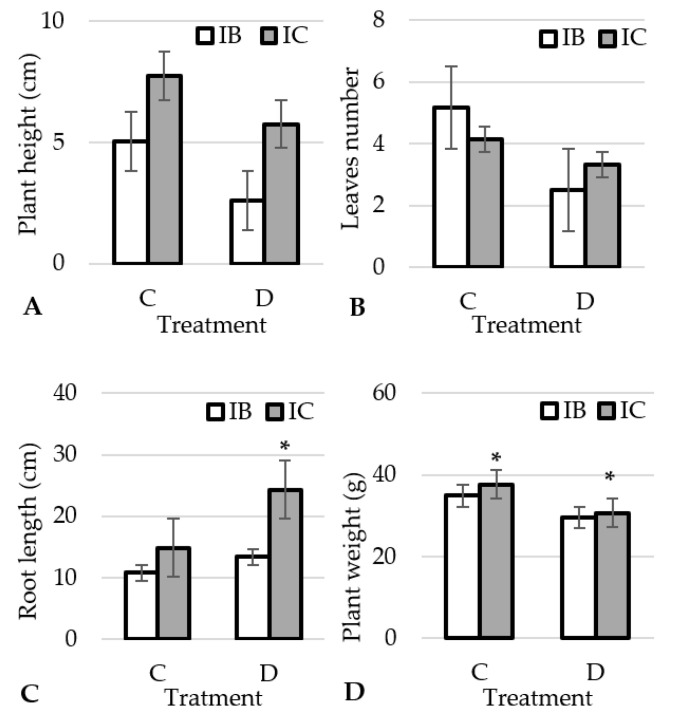
Effect of treatments on plant height (**A**), number of leaves (**B**), root length (**C**) and plant weight (**D**) in garlic ecotypes. *-significant differences (*p* < 0.05) among the ecotype within each treatment. IB: Istarski bijeli, IC: ’Istarski crveni’, C: control, D: drought.

Significant differences in morphological characteristics were determined between the two studied garlic ecotypes in root length and plant weight. Drought treatment also had significant effects on all morphological characteristics (Table 1). Drought reduced plant height, number of leaves and plant weight (Figure 1A,B,D, respectively) and increased root length (Figure 1C) in both ecotypes, however this increase in root length was more pronounced in the drought treatment in the ecotype IC than in IB. The plants develop deeper roots to access moisture and maintain a balance between root water uptake and the photosynthetic activities of the aerial part.

These results are supported by the research of Chaudhry et al. [13], who reached a similar conclusion in onions (*Allium cepa* L.). In their study, all onion cultivars tested showed increased root length development under drought stress. They suggest that the increase in root length is due to the ability to mitigate osmotic stress by maintaining osmotic potential. Plant weight was higher in ecotype IC than in IB in both treatments (Figure 1D). In our study, the interaction between ecotype and treatment was significant only for the number of leaves (Table 1). The reduction in the number of leaves during drought is the result of the plants primary drought defence mechanisms. By reducing leaf area and decreasing the number of leaves, the plant conserves water by reducing the area for transpiration [29]. Lack of water leads to a decrease in turgor, cell growth and division are inhibited, resulting in weaker leaf growth and faster senescence and shedding [30].

A decrease in plant height with increasing drought intensity in garlic was also determined by Marostica et al. [31]. The same result was obtained by Ajayi et al. [32] in amaranth and Hanci and Cebeci [33] in onion. In both studies, drought stress was found to significantly affect plant height and other morphological characteristics.

### 2.2. Chlorophyll Fluorescence and Physiological Characteristics of Garlic

Significant differences in leaf spectral characteristics were found between the two studied garlic ecotypes (Table 1).

Namely, higher reflection in red, green and blue and higher VAL were observed in IB compared to IC, while higher values of vegetation indices related to chlorophyll content (CHI, GI) and anthocyanin content (ARI) and VAL were observed in the ecotype IC (Supplementary Table S1). In addition, the ecotype IC exhibited higher minimum (F_0_) and maximum fluorescence (F_m_) compared to IB. However, this increase was proportional and therefore did not affect the final F_v_/F_m_ values, which were not significantly different between these two ecotypes.

Sperdouli et al. [34], according to Moustakas et al. [35], found in *Arabidopsis thaliana* that the intensity of stress had a significant effect on the maximum efficiency of PSII photochemistry (F_v_/F_m_). In this study, the maximum efficiency of PSII photochemistry (F_v_/F_m_) decreased 24 h after the beginning of drought stress, while it further decreased on the sixth day after the beginning of stress. However, ten days after the beginning of the stress, the maximum efficiency of PSII photochemistry recovered. Further drought treatment was severe for *Arbidopsis* and resulted in significantly decreased PSII [35]. PSII. Most authors did not find significant decreases in F_v_/F_m_ under moderate drought stress [36,37,38], suggesting that ETR is unaltered under drought stress [39]. These discrepancies in F_v_/F_m_ were mainly due to the differences among plant species, the stage of plant growth and development when drought occurs, the duration of the stressful period and their susceptibility to drought stress [40]. Here we hypothesize that the more pronounced effect of drought treatment on examined multispectral and chlorophyll fluorescence characteristics are absent due to the morphological and biochemical changes which probably had a protective role regarding physiological functions.

### 2.3. Influence of Drought on Biochemical Characteristics of Garlic

#### 2.3.1. Total Phenolic, Sugar and Inulin Contents and Antioxidant Activity

The response of plants to drought is complex and is reflected on the morphological, physiological and biochemical levels. Osmotic adjustment (OA), because of the accumulation of cellular compatible solutes, is an important adaptive mechanism to drought stress and is widely recognized to have a role in plant adaptation to dehydration mainly through turgor maintenance and the protection of specific cellular functions by defined solutes [41]. The well-known organic osmolytes include sugars, fructans (inulin) and free amino acids, especially proline. The substances mentioned are not only osmolytes but, together with phenolic compounds, act as ROS scavengers that reduce the oxidative stress that occurs as a result of primary abiotic stress. Higher levels of phenolic compounds are therefore associated with high antioxidant capacity in plants, including garlic [42,43,44]. To estimate the antioxidant capacity of plant extracts, several assays have been used, including DPPH (2,2-dyphenyl-1-picrylhydrazyl) and FRAP (ferric reducing antioxidant potential) [45]. Drought has different effects on the antioxidant activity of plant genotypes. One group of authors found an increase in antioxidant activity under the influence of drought [46,47], while another group found a decrease in antioxidant activity [48,49].

**Figure 2:**
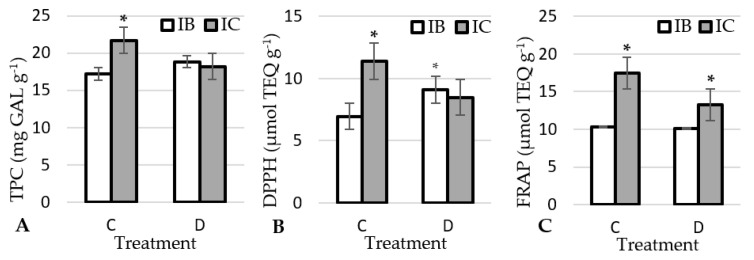
Effect of treatments on TPC (**A**), DPPH (**B**) and FRAP (**C**) in garlic ecotypes. * significant differences (*p* < 0.05) among the ecotype within each treatment. IB: Istarski bijeli, IC: ‘Istarski crveni’, C: control, D: drought.

This study determined significant differences in total phenolic content (TPC) and antioxidant activity between the two studied garlic ecotypes. The interaction between ecotype and treatment was also significant (Table 2). In the control treatment, the ecotype IC was found to have a higher total phenolic content as well as a higher antioxidant capacity as measured by DPPH radical scavenging activity and FRAP. Under drought conditions, higher DPPH was determined in IB and higher values of FRAP in IC ecotype (Figure 2A–C).

Some authors have reported that accumulation of phenolic compounds in plants under abiotic stress leads to an increase in antioxidant activity [50,51]. In contrast, Astaneh et al. [52] showed that garlic loses phenolic compounds under drought induced by salinity, as evidenced by phenol leakage and lower total phenolic content of the stressed plants compared to the control. In a subsequent study, the same authors reported a decrease in antioxidant capacity, measured as DPPH, of garlic plants grown under drought induced by salinity [52]. Phenolic compounds play a key role in radical scavenging activity. However, since the content of phenolic compounds under drought is the same for both ecotypes in this study, the differences in antioxidant capacity measured by DPPH and FRAP under drought treatment are probably caused by differences in other antioxidant compounds such as sugars and/or inulin. Regardless, a very strong, significant correlation (average of both treatments) was determined between antioxidant capacity, measured by DPPH and FRAP, and TPC in the garlic ecotypes. The positive correlation between TPC and antioxidant activity was observed in cereals [53], soybean [54] and lettuce seedlings [55], suggesting that the degradation of DPPH and FRAP largely depends on the presence of large amounts of phenolic compounds [56].

**Figure 3:**
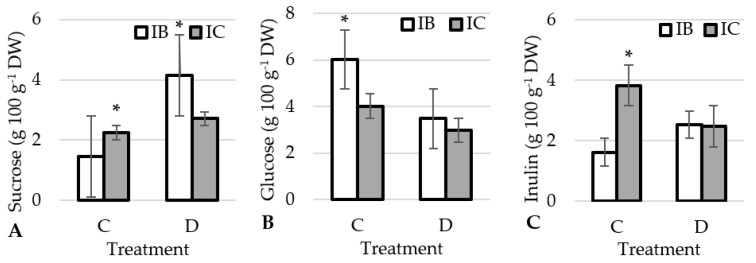
Effect of treatments on sucrose (**A**), glucose (**B**) and inulin (**C**) in garlic ecotypes. *-significant differences (*p* < 0.05) among the ecotype within each treatment. IB: Istarski bijeli, IC: ‘Istarski crveni’, C: control, D: drought.

A significant difference in sugar content was determined between ecotypes and treatments. The interaction between ecotypes and treatment was also significant (Table 2). Higher sucrose content was determined in the control treatment in ecotype IC (Figure 3A), while ecotype IB had higher sucrose content in drought and higher glucose content in both treatments (Figure 3A,B).

The reason for the lower accumulation of sucrose in the drought treatment in the ecotype IC compared to IB could be a better translocation of sucrose into the root during drought and thus a stronger root growth, and IC indeed had much longer roots in drought than in the control (Figure 1C). The deep root system is adapted for absorbing deep soil water under extreme drought conditions [57]. Since the major vegetative organs are underground, the plant root system relies on translocation of photoassimilates to sustain growth. Sucrose is the most common form of transported photoassimilate that is transported by phloem from the source tissue (e.g., shoots) to the sink tissue (e.g., roots) [58]. As Durand et al. [59] and Chen et al. [60] found in *Arabidopsis*, key players in phloem loading and sucrose transport from the shoot to the root are the sucrose transporters SUT and SWEET. The SWEET11 and 12 transporters in *Arabidopsis* are rapidly phosphorylated under drought and abscisic acid treatment. This phosphorylation enhances the oligomerization and sucrose transport activity of SWEETs, resulting in increased sucrose content in roots, improved root growth under drought and drought resistance [60]. Importantly, this enhanced root growth did not compromise shoot growth, as can be seen from plant height, number of leaves and plant weight of IC under drought stress (Figure 1A,B,D). Drought reduces the intensity of photosynthesis and reduces CO_2_ uptake, but does not significantly reduce respiration, so metabolism requires more soluble sugars.

**Figure 4:**
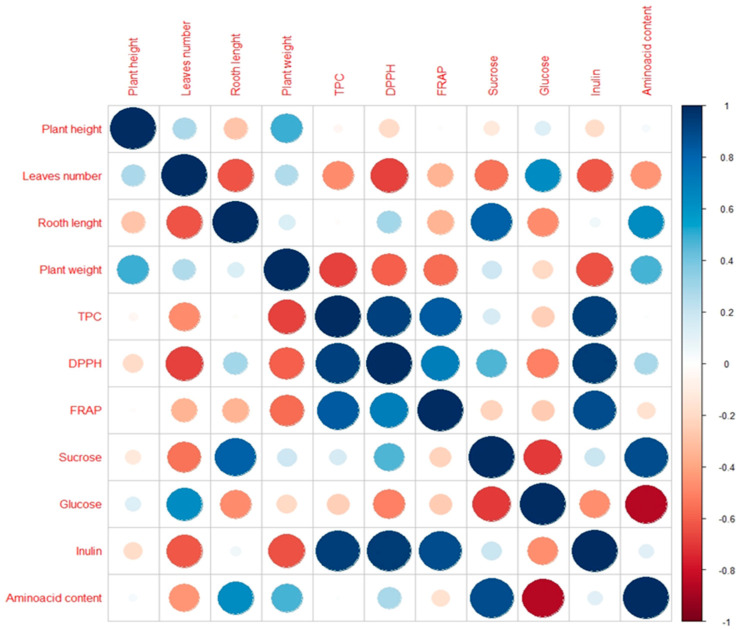
Correlation analysis for morphological characteristics, total phenolic content, antioxidant capacity, sugar, inulin, and amino acid content. Blue and red dots represent positive and negative correlation, respectively. Colour intensities are proportional to the correlation coefficients, as shown in the legend to the right.

Soluble sugars trigger the proliferation of organs and produce larger and thicker leaves [61]. In this study, glucose content showed a significant positive correlation with the number of leaves and plant height (Figure 4). This especially applies to ecotype IB in the control treatment (Figure 1B). At the same time, there was no correlation with plant weight. In this study, the drought treatment reduced the glucose content in the leaves in both ecotypes (Figure 3B). This is in agreement with findings of Hlahla et al. [62] in vegetable-type soybean. The content of the monosaccharide glucose was reduced in favour of the disaccharide sucrose, which is confirmed by the significant negative correlation between glucose and sucrose (Figure 4). Soluble sugars are required for osmotic regulation to maintain phloem turgor and to maintain phloem transport under drought stress [63]. However, a high concentration of soluble sugars reverses physiological processes in a concentration-dependent manner [64] and inhibits photosynthesis as a result of sugar accumulation [65].

Increased accumulation of inulin could protect cells from dehydration during drought [66]. The protective action of fructans can be attributed to their ability to insert themselves between the lipids of the membrane [66], and thus stabilize it during drought [67]. Vereyken et al. [68] demonstrated that inulin-type fructans protect membranes. Besides this, fructans have also been reported to have antioxidant properties [69]. Fructans, as well as other sugars, have been shown to be better scavengers of ◦OH radicals compared to O_2_^−^ [70]. Since plants lack enzymatic ◦OH-scavenging mechanisms, high concentrations of non-enzymatic antioxidant mechanisms are used to neutralize ROS [71]. In this study, the ecotype IC had a significantly higher inulin content in the control, but in drought conditions IC reduced the inulin content. In contrast, IB showed a tendency to increase inulin content under drought conditions (Figure 3C). The antioxidant activity of inulin in garlic is obviously very high, as indicated by the very high correlation coefficients of inulin with DPPH and FRAP (Figure 4). Based on the results obtained, the antioxidant properties of the ecotype IB in drought treatment are not only the result of TPC but also of inulin content.

#### 2.3.2. Amino acid Compounds

Free amino acids act as osmoprotectants, and can function as ROS scavengers (e.g., Pro) [24], but also have an ameliorating role in drought-induced nitrogen uptake, through its reassimilation and maintenance of protein homeostasis [72]. In situations of insufficient carbohydrate supply due to a decrease in photosynthesis rates, which usually occur during stress conditions, plants can use amino acids as alternative substrates for mitochondrial respiration [24].

In this study, ecotype, treatment and ecotype x treatment interaction significantly affected amino acid content (except for proline, histidine, threonine and alanine) (Table 2). In both ecotypes, IC and IB, an increase in almost all amino acids is observed in plants exposed to drought compared to the control, which is a strong signal that the plants are suffering from drought (Supplementary Table S2). Numerous studies have established a significant increase in amino acid content under drought [23,73,74,75,76]. Namely, drought increases proteolysis in plants and consequently increases the content of free amino acids and their metabolites [75].

Proline (Pro) synthesis in plants is a common physiological response to drought. Pro plays a role as an osmolyte, antioxidant, chaperone, stabilizer of membranes and enzymes [76]. However, an extreme increase in proline is not good for plants because it comes at the cost of other amino acids [75]. Schafleitne et al. [77] found higher proline accumulation in drought-susceptible than in drought-resistant potato genotypes. In contrast, Ghodke [78] found that a higher increase in proline levels reflects the adaptation mechanism to drought present in the tolerant onion genotype compared to the drought-sensitive genotype. In both ecotypes studied here, Pro content in drought treatment was about twice as high as in plants exposed to control treatment (Table 3), indicating a similar stress response in these two ecotypes.

The upregulation of genes related to tyrosine, glycine, serine and threonine metabolism in garlic under drought was found by Zhou et al. [25]. Our results are in partial agreement with those of Zhou et al. [25]. Significantly higher levels of serine and threonine were found under drought in both ecotypes, but the levels of tyrosine and glycine did not change significantly under drought in IC (Table 3). Apart from these two amino acids, drought had no effect on the content of methionine, phenylalanine, isoleucine and leucine in IC. In the ecotype IB, on the other hand, the content of isoleucine and leucine was significantly higher under drought compared to the control treatment. These two amino acids belong to the branched-chain amino acids that have been shown to be associated with drought-tolerant genotypes of potato [73] and tomato [72]. A significant positive correlation was found between total free amino acid content and root length (Figure 4).

## 3. Materials and Methods

### 3.1. Plant Material and Growth Conditions

In this study, garlic cloves (*Allium sativum* L.) of ‘Istarski crveni’ (IC) and Istarski bijeli (IB) ecotypes provided by the Institute for Agriculture and Tourism Poreč were used. The cultivation of plants was carried out in the growth chambers at the Faculty of Agriculture, University of Zagreb. Cloves were treated with POLYRAM ^®®^ DF (Chromos-agro, Zagreb, Croatia) fungicide before planting. Individual cloves were planted in a substrate of a mixture of black and brown peat Kekkila TSM 3 (Kekkila Proffesional, Vantaa, Finland) and placed in the growth chamber. The temperature in the chamber was 22 °C, the relative humidity of 50%, and the photoperiod was 16/8 h (light/darkness) and light intensity 250 µmol m^−2^ s^−1^ provided by PhenoLight3 (PhenoVation, Wageningen, The Netherlands). A total of 50 IC cloves and 50 IB cloves were planted. After the initial growth, before inducing drought stress, plants of the same habitus (equal height, number of leaves, good general condition) were selected. A total of 54 plants were used in the experiment, of which 30 were IC and 24 plants were IB. In each treatment, 15 IC plants and 12 IB plants were tested.

The drought stress induction started 21 days after clove planting when plants had three developed leaves. The experiment was carried out in two treatments: (1) Control (C)-plants were watered twice a week with tap water; (2) Drought stress (D)-plants were watered with a 15% solution of polyethylene glycol 8000 (PEG) (−0.6 MPa) twice a week. Drought stress was induced for 20 days.

### 3.2. Morphological Analysis

Morphological measurements were performed 20 days after the induction of drought stress. The following morphological characteristics were analysed: plant height (the length of the bulb together with above ground part, cm), the number of leaves, root length (the length from the end of the bulb to the tips of the roots using a ruler, cm) and plant fresh weight (g).

### 3.3. Chlorophyll Fluorescence and Multispectral Analysis

Chlorophyll fluorescence and multispectral analysis were performed at the end of stress induction period (41 days after planting). All plants were recorded using a CropReporter^TM^ (PhenoVation B.V., Wageningen, The Netherlands) device. All images are captured with the same lens (10 Mp lens, 200 Lp mm^−1^ resolution, 400–1000 nm spectral range) and CCD-camera (1.3 Mp, 1296 × 966 pixels), with real 14-bit signal resolution. The output is 16-bit RAW format, and automatic analysis of chlorophyll fluorescence, colour, and multispectral images was performed by DA TM software (PhenoVation B.V., Wageningen, The Netherlands). For chlorophyll fluorescence analysis, plants were imaged using an optimized slow fluorescence induction protocol [79], which includes dark adaptation, measuring the induction curve of dark-adapted plants followed by turning on the light for light adaptation and measuring the induction curve of light-adapted plants. For chlorophyll fluorescence measurements of dark-adapted plants (plants were dark adapted for 30 min), a saturating pulse of light of 4000 µmol m^−2^ s^−1^ for 800 ms was used. The minimum chlorophyll fluorescence (F_0_) was measured after 10 µs, and the maximum chlorophyll fluorescence (F_m_) was measured after saturation. After measuring the dark-adapted plants, the plants were left in the dark for 15 s, and then the actinic lights (300 µmol m^−2^ s^−1^) were turned on to allow the plants to light-adapt for 5 min. Steady-state fluorescence yield (F_s_′) was measured at the beginning of the saturation pulse, and maximum chlorophyll fluorescence (F_m_′) of light-adapted plants was measured at saturation, using saturation pulse intensity (4000 µmol m^−2^ s^−1^).

After the measurement, actinic light was turned off, and in the presence of far-red light, the minimal fluorescence yield of the illuminated plant (F_0_′) was estimated. These measured parameters were used for the calculation of different chlorophyll fluorescence parameters, which are shown in Table 4.

Following chlorophyll fluorescence imaging, under actinic light (300 µmol m^−2^ s^−1^) images of spectral reflectance were collected in red (RRed—640 nm), green (RGreen—550 nm), blue (RBlue—475 nm), specific green (RSpcGrn—510–590 nm), chlorophyll reflectance (RChl—730 nm) near infra-red (RNIR—769 nm), and far-red (RFarRed—710 nm) reflectance. From measured reflectance normalized difference vegetation index (NDVI) [83], chlorophyll index (CHI) [83], anthocyanin index (ARI) [84], hue (0–360°), saturation (SAT), and value (VAL) were calculated.

A list of all measured multispectral parameters and calculated vegetation indices are given in Table 5.

### 3.4. Biochemical Analysis

Plant leaves for analysis of biochemical parameters were collected 20 days after induced drought stress from all plants included in each treatment. The lyophilized plant tissue was milled to a particle size of 0.2 mm on a centrifugal mill (Retsch ZM-200, Haan, Germany). Samples (50 mg) were homogenized with 2.4 mm glass beads (Omni kit 19-670, Kennesaw, GA, USA) for 1 min at 5 ms^−1^ in 1.5 mL of aqueous methanol (80:20, methanol: water, *v*/*v*) using a bead mill (Omni Bead Ruptor Elite, Kennesaw, GA, USA). The samples were left to macerate for 1 h on a rotator (Biosan RS-60, Riga, Latvia) and subsequently centrifuged for 5 min at 16,000× *g*. The supernatants were filtered through a 0.22 µm nylon filter prior to analysis.

#### 3.4.1. Total Phenolic Content

The total phenolic content (TPC) was determined according to Singleton and Rossi [86]. Briefly, 20 µL of the sample was mixed with 140 µL of 0.2 M Folin–Ciocalteu reagent, and, after 1 min, 140 µL of 6% sodium carbonate was added. The reaction mixture was incubated at 25 °C for 1 h and the absorbance was read at 750 nm (Tecan Infinite 200 Pro M Nano+, Männedorf, Switzerland). The TPC was standardised against the gallic acid and expressed as the mg of gallic acid equivalents per g sample in DW. The results were calculated against a standard curve of gallic acid (serial dilutions of gallic acid: 12.5, 25, 50, 75, 100, 150 and 250 mg L^−1^) and expressed as mg GAEQ g^−1^ DW. Determined R^2^ was 0.9999.

#### 3.4.2. Total Antioxidant Activity

The total antioxidant activity was evaluated using the FRAP assay (ferric-reducing antioxidant capacity) [87] and the DPPH radical scavenging activity (2,2-diphenyl-1-picrylhydrazyl) assay [88]. Briefly, 100 µL of the sample was mixed with 200 µL of either freshly prepared FRAP reagent or 0.02 M DPPH radical for the FRAP or DPPH assays, respectively. The antioxidant capacity using the FRAP assay was evaluated by reading the absorbance at 593 nm (Tecan Infinite 200 Pro M Nano+, Männedorf, Switzerland) after 10 min of reaction time at 25 °C, while the DPPH radical scavenging capacity was evaluated by reading the absorbance at 517 nm (Tecan Infinite 200 Pro M Nano+, Männedorf, Switzerland) after 30 min of reaction time at 25 °C. Both the FRAP and DPPH values were calculated against a standard Trolox calibration curve (serial dilutions of Trolox—2, 5, 10, 25, 50, 75 and 100 µM) and expressed as µmol Trolox g^−1^ DW. Determined R^2^ were 0.9998 and 0.9997, respectively.

#### 3.4.3. Sugar and Inulin Content

Sucrose, glucose and fructooligosaccharide (expressed as inulin) contents were analysed according to Major et al. [89] using an HPLC system consisting of a system controller (Shimadzu CBM-40, Kyoto, Japan), a degassing unit (Shimadzu DGU-405, Kyoto, Japan) a solvent delivery unit (Shimadzu LC-20Ai, Kyoto, Japan), an autosampler (Shimadzu SIL-20AC, Kyoto, Japan), column oven (Shimadzu CTO-40S, Kyoto, Japan) and a refractive index detector (Shimadzu RID-20A, Kyoto, Japan). Chromatographic separation was achieved by injecting 10 µL of the sample on a 300 × 8 mm, 9 µm particle size, calcium cation exchange column (Dr. Maisch ReproGel Ca, Ammerbuch, Germany) held at 80 °C using deionized water as the mobile phase (0.6 mL/min, isocratic elution). Retention times and peak areas of the investigated sugars were compared to analytical standards for identification and quantification, respectively. Linear calibration curves were obtained with serial dilutions of 0.1, 0.5, 1.0, 5.0, 10.0 g L^−1^ of inulin (coefficient of determination, R^2^ = 0.99999), sucrose (coefficient of determination, R^2^ = 0.99999), and glucose (coefficient of determination, R^2^ = 0.9999).

#### 3.4.4. Free Amino Acids

The free amino acids were analysed by HPLC consisting of a binary solvent delivery unit (Shimadzu LC-40D X3, Tokyo, Japan), a degassing unit (Shimadzu DGU-40, Tokyo, Japan), an autosampler (Shimadzu SIL-40C, Tokyo, Japan), a column oven (Shimadzu CTO-40C, Tokyo, Japan) and a fluorescence detector (Shimadzu FLD20, Tokyo, Japan). The amino acids were analysed according to Kralj Cigić et al. [90] with some modifications. Briefly, the amino acids were derivatized by pre-column derivatization of the primary and secondary amino acids by o-phthaldialdehyde (OPA)/3-mercaptopropionic acid (MPA) and 9-Fluorenylmethoxycarbonyl chloride (FMOC) reagents, respectively. The derivatization was performed by the autosampler. The derivatized amino acids were separated by injecting 5 µL of the mixture on an octadecylsilane column, 150 × 2.1 mm, 2.7 µm particle size (Agilent Poroshell HPH C18, Santa Clara, CA, USA) maintained at 40 °C with a gradient elution of mobile phase A (borate buffer, pH 8.2) and mobile phase B (acetonitrile: methanol: water—45:45:10—*v*/*v*/*v*) in the following program: 0–12 min, 2%B–57%B; 12–14 min, 57%B–100%B; 14–16 min, 100%B, 16–17 min, 100%B–2%B; 17–20 min, 2%B flow rate. The primary and secondary acids were monitored on the excitation/emission wavelengths of 340/450 nm and 260/305 nm, respectively. The amino acids were identified and quantified by the retention times and areas under the curve of serial dilutions of reference standards.

### 3.5. Statistical Analysis

All experiments were set up in a completely randomized design. The normality of the residuals for each model were assessed by visual inspection of the diagnostic plots as suggested by Kozak and Piepho [91]. The data from all measurements were subjected to ANOVA. The Bonferroni post-hoc test at *p* ≤ 0.05 was used for means comparison. Correlation analysis was performed using RStudio (ver. 5.2; RStudio Desktop, Boston, MA, USA) at *p* < 0.05. The other statistical analysis of the data was carried out using the SAS/STAT^®®^ [92] program package.

## 4. Conclusions

Based on the results obtained, we determined that drought had a significant effect on the morphological characteristics of both ecotypes of garlic. While plant height, number of leaves and plant weight decreased, root length increased in drought compared to the control treatment. Although significantly higher plant weight was observed at IC, this is influenced by the genotype rather than the environment, since this ecotype also had higher weight in the control treatment. However, no significant influence of drought was observed in the physiological characteristic, except for CHI, F_v_/F_m_ and F_0_. Apparently, the ecotypes were able to overcome the moderate drought stress without major damage to the photosystem due to their excellent antioxidant potential and the increase in the content of osmoprotectants. The garlic ecotypes responded differently to drought in terms of biochemical parameters. The drought tolerance of IB could be related to increased accumulation of inulin and higher levels of amino acids, especially those shown to contribute to drought resistance. In IC, drought tolerance is associated with an increase in some amino acid compounds and a decrease in the leaves’ sucrose content due to translocation to the underground part of the plant to intensify root growth at depth.

## Figures and Tables

**Table 1:** Analysis of variance of morphological and physiological characteristics, and chlorophyll fluorescence parameters of garlic.

Characteristics	Ecotype (E)		Treatment (T)		ExT	
	F Value	Pr > F	F Value	Pr > F	F Value	Pr > F
Plant weight	22.03	***	12.97	**	0.13	ns
Plant height	0.48	ns	4.81	*	0.09	ns
Number of leaves	0.06	ns	19.16	**	5.42	*
Root length	20.23	**	13.11	**	4.27	ns
RED	10.1	**	8.19	**	0.03	ns
GREEN	5.61	*	3.04	ns	0.13	ns
BLUE	9.66	**	0.88	ns	0.50	ns
FAR RED	2.61	ns	3.65	ns	0.10	ns
NIR	0.01	ns	0.62	ns	1.75	ns
HUE	2.07	ns	2.43	ns	0.55	ns
SATURATION	4.78	*	0.37	ns	0.40	ns
VALUE	5.87	*	3.61	ns	0.09	ns
CHI	4.70	*	14.01	**	1.74	ns
ARI	7.89	**	0.17	ns	1.66	ns
NDVI	0.81	ns	1.18	ns	3.40	ns
GI	9.35	**	0.19	ns	0.02	ns
F_0_	6.57	*	1.05	ns	0.83	ns
F_v_/F_m_	0.18	ns	19.07	***	2.24	ns
F_s_	2.07	ns	0.50	ns	0.38	ns
F_m_	10.32	**	10.42	**	1.66	ns
F_q_/F_m_	0.26	ns	0.88	ns	0.06	ns
ETR	2.64	ns	3.16	ns	2.97	ns
NPQ	0.96	ns	3.08	ns	5.25	*

*, **, and *** indicate significance at *p* < 0.05, *p* < 0.01, and *p* < 0.001, respectively. ns: non-significant.

**Table 2:** Analysis of variance of biochemical characteristics of garlic.

Biochemical Characteristics	Ecotype (E)		Treatment (T)		ExT	
	F Value	Pr > F	F Value	Pr > F	F Value	Pr > F
TPC	11.89	**	2.96	ns	21.79	**
DPPH	29.43	**	0.06	ns	114.72	***
FRAP	183.03	***	34.09	**	27.19	**
Sucrose	15.53	**	337.42	***	189.61	**
Glucose	101.50	***	208.48	***	37.39	**
Inulin	187.43	***	7.81	*	205.19	***
Proline	1.41	ns	379.49	***	12.67	**
Asparagine	56.77	***	709.33	***	9.80	*
Glutamine	19.53	**	776.50	***	16.00	**
Serine	17.43	**	333.61	***	133.06	***
Histidine	0.96	ns	233.27	***	28.06	**
Glycine	50.86	***	245.36	***	189.65	***
Threonine	1.19	ns	484.76	***	95.22	***
Arginine	67.66	***	1219.80	***	48.90	***
Alanine	1.53	ns	188.26	***	57.39	***
Tyrosine	92.22	***	175.36	***	249.14	***
Methionine	30.47	**	31.34	**	62.70	**
Phenylalanine	79.91	***	91.22	***	118.61	***
Isoleucine	119.20	***	183.18	***	177.72	***
Leucine	104.04	***	102.83	***	144.31	***
Lysine	28.26	**	242.99	***	53.13	***
AA	7.52	*	547.92	***	73.32	**

*, **, and *** indicate significance at *p* < 0.05, *p* < 0.01, and *p* < 0.001, respectively. ns: non-significant. AA: total content of all analysed free amino acids.

**Table 3:** Effect of drought stress on amino acid content in garlic ecotypes.

Amino Acid (µmol 100 g^−1^ DW)	IB		IC	
	*C*	*D*	*C*	*D*
Proline	0.79	1.66 *	0.79	1.57 *
Asparagine	1.85	6.06 *	3.36	6.68 *
Glutamine	2.66	8.96 *	4.32	9.05 *
Serine	2.62	7.55 *	5.22	6.33 *
Histidine	0.33	0.62 *	0.39	0.53 *
Glycine	0.69	1.63 *	0.90	0.96
Threonine	0.56	1.61 *	0.85	1.25 *
Arginine	1.42	4.91 *	1.57	6.82 *
Alanine	1.29	2.52 *	1.80	2.15 *
Tyrosine	0.24	0.54 *	0.30	0.28
Methionine	0.22	0.31 *	0.23	0.22
Phenylalanine	0.27	0.46 *	0.29	0.27
Isoleucine	0.27	0.56 *	0.29	0.30
Leucine	0.54	0.97 *	0.57	0.53
Lysine	0.97	1.88 *	1.05	1.88 *
AA	15.21	41.51 *	22.66	39.07 *

* indicates significance at *p* < 0.05 among the treatments within each ecotype. C: control, D: drought, IB: Istarski bijeli, IC: ‘Istarski crveni’. AA: total content of all analysed free amino acids.

**Table 4:** Chlorophyll fluorescence parameters, the equation for calculation, and the reference.

Abbrev	Trait	Wavelength/Equation
F_v_/F_m_	Maximum Efficiency of PS II	F_v_/F_m_ = (F_m_ − F_0_)/F_m_ [80]
F_q_′/F_m_′	Effective Quantum Yield of PS II	F_q_′/F_m_′ = (F_m_′ − F_s_′)/F_m_′ [81]
ETR	Electron Transport Rate	ETR = F_q_′/F_m_′ × PPFD × (0.5) [81]
NPQ	Non-Photochemical Quenching	NPQ = (F_m_ − F_m_′)/F_m_′ [82]

**Table 5:** List of analysed multispectral parameters, wavelength for measurement or equation for calculation, and the reference.

Abbrev	Trait	Wavelength/Equation
R_Red_	Reflectance in Red	640 nm
R_Green_	Reflectance in Green	550 nm
R_Blue_	Reflectance in Blue	475 nm
R_SpcGrn_	Reflectance in Specific Green,	510–590 nm
R_FarRed_	Reflectance in Far Red	710 nm
R_NIR_	Reflectance in Near Infra-Red	769 nm
R_Chl_	Reflectance Specific to Chlorophyll	730 nm
HUE	Hue (0–360°)	HUE = 60 × (0 + (R_Green_ − R_Blue_)/(max − min)), if max = R_Red_; HUE = 60 × (2 + (R_Blue_ − R_Red_)/(max − min)), if max = R_Green_; HUE = 60 × (4 + (R_Red_ − R_Green_)/(max − min)) if max = R_Blue_; 360 was added in case HUE < 0
SAT	Saturation (0–1)	SAT = (max − min)/(max + min) if VAL > 0.5, or SAT = (max − min)/(2.0 – max − min) if VAL < 0.5, where max and min are selected from the R_Red_, RG_reen_, R_Blue_
VAL	Value (0–1)	VAL = (max + min)/2; where max and min are selected from the R_Red_, R_Green_, R_Blue_
ARI	Anthocyanin Index	ARI = (R_550_)^−1^ − (R_700_)^−1^ [85]
CHI	Chlorophyll Index	CHI = (R_700_)^−1^ − (R_769_)^−1^ [84]
NDVI	Normalized Differential Vegetation Index	NDVI = (R_NIR_ − R_Red_)/(R_NIR_ + R_Red_) [83]

## Data Availability

The data is contained within the manuscript and Supplementary Materials.

## Supplementary Materials

The following supporting information can be downloaded at: https://www.mdpi.com/article/10.3390/plants12091824/s1, Table S1. Average values of morphological and physiological characteristics, and chlorophyll fluorescence parameters from main factors, Table S2. Average values of biochemical characteristics from main factors.
