# Supplementary material for: Garlic Ecotypes Utilise Different Morphological, Physiological and Biochemical Mechanisms to Cope with Drought Stress
Source: Plants (Basel). 2023 Apr 28;12(9):1824. doi: 10.3390/plants12091824 (PMC10180593; doi:10.3390/plants12091824)
Supplement: Supplementary file 1 [file plants-12-01824-s001.zip › plants-2287588-supplementary.pdf]

plants

MDPI

Supplementary Materials

Table S1. Average values of morphological and physiological characteristics, and chlorophyll fluorescence parameters from main factors.

Characteristic	Genotype		Treatment	
	**IB**	**IC**	**C**	**D**
Plant height (cm)	32.27	34.22	36.32*	30.17
Leaves number	3.83	3.74	4.65*	2.92
Rooth length (cm)	12.11	19.58*	12.84	18.86*
Plant weight (g)	3.82	6.75*	6.39*	4.18
Red	1446*	1279	1287	1438*
Green	2100*	1933	1955	2078
Blue	1451*	1255	1324	1383
Far red	3437	3267	3252	3452
NIR	11633	11611	11742	11502
HUE	129	125	129	125
SAT	0.37	0.41*	0.39	0.40
VAL	0.032*	0.029	0.030	0.032
CHI	2.48	2.61*	2.65*	2.43
ARI	1.97	2.19*	2.10	2.07
NDVI	0.63	0.64	0.64	0.62
GI	0.26	0.31*	0.28	0.29
F_0	1652	1825*	1773	1704
F_v/F_m	0.83	0.83	0.84*	0.82
F_s	2925	3093	3051	2968
F_m	9961	11025*	11027*	9959
F_q/F_m	0.47	0.48	0.48	0.47
ETR	11491	12176	12208	11459
NPQ	0.87	0.92	0.95	0.84

* indicate significance at $p < 0.05$ among the genotypes and treatments. C: control, D: drought, IB: Istarski bijeli, IC: 'Istarski crveni'.

Table S2. Average values of biochemical characteristics from main factors.

Characteristic	Genotype		Treatment	
	IB	IC	C	D
TPC (mg GAL g^{-1} DW)	18.02	19.92*	19.46	18.50
DPPH (μmol TEQ g^{-1} DW)	8.43	9.89*	9.13	9.20
FRAP (μmol TEQ g^{-1} DW)	10.23	15.37*	13.98*	11.69
Sucrose (g 100 g^{-1} DW)	2.80*	2.48	1.85	3.43*
Glucose (g 100 g^{-1} DW)	4.74*	3.50	5.02*	3.23
Inulin (g 100 g^{-1} DW)	2.07	3.15*	2.72*	2.50
Proline	1.22	1.27	0.88	1.61*
Asparagine	3.95	5.02*	2.60	6.37*
Glutamine	5.81	6.69*	3.49	9.01*
Serine	5.09	5.76*	3.92	6.94*
Histidine	0.48	0.46	0.36	0.58*
Glycine	1.16*	0.93	0.80	1.29*
Threonine	1.09	1.05	0.71	1.43*
Arginine	3.17	4.20*	1.50	5.87*
Alanine	1.90	1.98	1.55	2.33*
Tyrosine	0.39*	0.29	0.27	0.41*
Methionine	0.26*	0.22	0.22	0.27*
Phenylalanine	0.37*	0.28	0.28	0.37*
Isoleucine	0.41*	0.30	0.28	0.43*
Leucine	0.75*	0.55	0.55	0.75*
Lysine	1.42*	1.21	1.01	1.63*
AA	28.36	30.87*	18.94	40.29*

* indicate significance at $p < 0.05$ among the genotypes and treatments. C: control, D: drought, IB: Istarski bijeli, IC: 'Istarski crveni', AA: total content of all analysed free amino acids.
